# Can sexual transmission support the enzootic cycle of *Trypanosoma cruzi*?

**DOI:** 10.1590/0074-02760170025

**Published:** 2018-01

**Authors:** Adriano Rios, Marcelle Ribeiro, Alessandro Sousa, Fernando Pimentel, Luciana Hagström, Rafael Andrade, Rozeneide M Alves, Ana de Cássia Rosa, Antônio RL Teixeira, Nadjar Nitz, Mariana M Hecht

**Affiliations:** Universidade de Brasília, Faculdade de Medicina, Laboratório Interdisciplinar de Biociências, Brasília, DF, Brasil

**Keywords:** Trypanosoma cruzi, sexual transmission, enzootic cycle

## Abstract

**BACKGROUND:**

*Trypanosoma cruzi* circulates in sylvatic habitats, mainly through blood-feeding triatomines, although other routes also contribute to its dispersion. Sexual transmission of *T. cruzi* is an understudied topic, especially among wild mammals. Because of the difficulties inherent to field work, experimentally infected mice are frequently used to evaluate the transmission of *T. cruzi*.

**OBJECTIVE:**

This study aimed to evaluate the sexual transmission of *T. cruzi* in acutely infected mice.

**METHODS:**

Male and female mice in the acute phase of Chagas disease were mated with naïve partners. Then, parasitological tests, immunohistochemistry, serological assays, and polymerase chain reaction (PCR) assays were used to detect infection.

**FINDINGS:**

Parasitological analysis showed trypomastigotes in the blood of 20% of the naïve mice after mating with infected partners. Serological assays detected anti-*T. cruzi* antibodies in all naïve females mated with infected males and in 60% of naïve males mated with infected females. PCR showed *T. cruzi* nDNA bands for all naïve mice mated with infected partners. The possibility of sexual transmission was also confirmed by visualisation of amastigotes in the testes.

**MAIN CONCLUSIONS:**

Our results demonstrate that sexual transmission of *T. cruzi* is an ordinary event that may contribute to maintenance of the parasite's enzootic cycle.

*Trypanosoma cruzi*, the etiologic agent of Chagas disease, infects hundreds of different mammalian species within eight orders (Teixeira et al. 2011). In hyper-endemic regions, marsupials, edentates, carnivores, and rodents are the most common sylvatic hosts (Teixeira et al. 2001, Yeo et al. 2005, Orozco et al. 2013). However, there is no record of a mammalian species that is refractory to *T. cruzi* infection, demonstrating that Chagas disease is a broad enzootic infection of wild animals.

Community diversity is a key factor in the circulation of many pathogens (Schofield 2000, Schmidt & Ostfeld 2001, Keesing et al. 2006), and the complex epidemiology of *T. cruzi* transmission within different ecosystems contributes to its ecological and evolutionary processes.

The flagellate protozoa *T. cruzi* is usually transmitted through infected faeces and/or urine excreted by triatomines (Hemiptera: Reduviidae) during blood feeding. However, this is a nonlinear phenomenon, as mammals can be exposed to infection multiple times through distinct routes (Jansen et al. 2015). The primitive oral route of protozoan acquisition is considered to be particularly common among skunks, armadillos, and anteaters (Teixeira et al. 2011), and it seems to be a highly efficient dispersion method (Yoshida 2009). Host-to-host dispersion, e.g., congenital transmission, is a vector-independent pathway that is also considered to be an important route in free-living wild mammals (Kribs-Zaleta 2014). Interestingly, it is believed that Chagas disease was established before hematophagy was acquired by triatomine bugs, and this acquisition was followed by a stepwise adaptation to vertebrate hosts (Teixeira et al. 2011).

Interestingly, it is believed that Chagas disease was established before hematophagy was acquired by triatomine bugs, and this acquisition was followed by a stepwise adaptation to vertebrate hosts (Teixeira et al. 2011). In this respect, opossums (*Didelphis sp*) are able to maintain amastigotes in tissues and epimastigotes in anal glands secretions, contaminating the enviroment (Lenzi et al. 1998). *T. cruzi* is a successful parasite that circulates in the bloodstream and is able to infect several tissue types in hundreds of different mammalian host species (Teixeira et al. 2011).

Many features of the enzootic transmission cycle of *T. cruzi* are unknown, as some routes in free-range mammals are poorly studied because of the difficulties inherent to field work. For example, there are no studies evaluating sexual transmission of *T. cruzi* in the wild. However, infected mice are commonly used as a model to evaluate and answer important questions in Chagas disease research as their physiology is similar to that of small sylvatic rodents (Krishnan et al. 2013). Some laboratory studies have demonstrated the possibility of sexual transmission of *T. cruzi* in mice (Alencar et al. 1991). However, two other studies in rodents obtained conflicting results; one showed that sexual transmission was rare (Martin et al. 2015), whereas another showed that it was an extremely frequent event (Ribeiro et al. 2016). Thus, further experiments are necessary to better understand *T. cruzi* dispersion via sexual intercourse.

## MATERIALS AND METHODS

*Parasite growth* - *T. cruzi* Berenice 78 trypomastigotes were maintained in L6 muscle cells (ATCC CRL-1458) and were used to infect mice. Epimastigotes were grown in liver infusion-tryptose medium for isolation of protozoan antigens and DNA (Ribeiro et al. 2016).

*T. cruzi infection* - Twenty, two-month-old, male and female BALB/c mice were bred and maintained in the Faculty Animal Housing Facility under positive air-pressure at 24°C, and were fed Purina chow and water *ad libitum*. Animals were allocated as follows: (I) five *T. cruzi-*infected males mated with five naïve females; and (II) five *T. cruzi-*infected females mated with five naïve males. Based on the recommendation of the Ethical Committee on Animal Research, uninfected, naïve mice were also used as a negative control group, and intraperitoneally infected mice were used as a positive control group to reduce the number of animals used in the study. To confirm the occurrence of sexual transmission, we also assessed 70 litters generated from groups I and II. Mice were infected by intraperitoneal injection of 1 × 10^5^ *T. cruzi* Berenice trypomastigotes.

*Parasitological assays for T. cruzi detection* - Fresh tail blood (5 μL) from intraperitoneally infected mice was diluted in saline and smeared on a glass slide. The smear was then examined for the presence of trypomastigotes under a microscope once a week for three weeks after infection. Additionally, three months after breeding, blood samples obtained from the mated mice by heart puncture were used for hemoculture. For the hemoculture, ~100 μL of heparinised blood was mixed with LIT medium and poured over a blood agar base in 50-mL glass tubes, which were incubated at room temperature. After 60 and 90 days of incubation, ~50 μL of the overlay the culture medium was examined under a microscope at 400×.

*Breeding T. cruzi-infected and uninfected mice and their progeny* - Five *T. cruzi-*infected females were bred with an equal number of uninfected male mice, and five *T. cruzi-*infected males were bred with an equal number of uninfected female mice. The mice were mated at three months of age and after complete healing of the tail, which was about ten days post blood collection. Each breeding pair was kept in a cage that was placed inside a safe box with a 5-mm grid and locking door to prevent escape. The cage was only taken out of the safe box for cleaning, one cage at a time. The mating couples were maintained in the same cage for approximately 15 days during female oestrus. After confirmation of pregnancy, the animals were kept in individual cages until euthanasia at six months of age. The mice were fed chow and water *ad libitum*. The parental (F0) and F1 progeny were inspected daily. Then, the negative and positive controls, as well the parents and F1 progeny, were bled via heart puncture to collect serum and nucleated cells. The F1 progeny were euthanised at one month old. All mice were euthanised by cervical dislocation under anaesthesia. Tissue sections from the heart and testes or ovary of F0 mice were embedded in paraffin and processed for pathological examination.

*DNA extraction and purification* - Total DNA was extracted from *T. cruzi* epimastigotes and from blood using the Biopur DNA Extraction MINI SPIN PLUS Kit (BIOPUR), according to the manufacturer's recommendations. The extracted DNA was quantified with a Nanovue (GE Life Sciences), and its quality was tested by PCR with *β-actin* primers. The amplification products were visualised by electrophoresis in agarose gels stained with ethidium bromide diluted in TAE buffer (90 mM Tris-acetate, pH 8.0, 25 mM EDTA). These standard procedures have been described elsewhere (Sambrook & Russel 2001).

*T. cruzi nuclear DNA (nDNA) amplification* - Molecular diagnosis of *T. cruzi* infection was performed in mating couples, progeny, and control mice by PCR using primers Tcz1 and Tcz2 (Moser et al. 1989) for amplification of a 188-bp fragment of parasite genomic DNA. Standard PCR procedures were used, and the reactions were run in triplicate, each containing 200 ng of *T. cruzi* DNA in 1× Invitrogen reaction buffer (20 mM Tris-HCl, pH 8.4, 50 mM KCl), 3 mM MgCl_2_, 0.1 μM each primer (Tcz1/2), 0.2 mM dNTPs (Illustra TM GE), and 2 U of *Taq* DNA Polymerase in a final volume of 25 μL. The reactions were run in a BIO-RAD *MyCycler TM* as described previously (Moser et al. 1989, Hecht et al. 2010).

*Southern blotting and hybridisation with radiolabelled probes* - The PCR products were separated in 1.3% agarose gels, and the DNA bands were transferred to positively charged nylon membranes (Hybond-XL; Amersham Pharmacia Biotech) by the alkaline transfer method (Sambrook & Russel 2001) via capillary action. The blot was dried at room temperature for DNA fixation. Then, *T. cruzi* nDNA and the fragments obtained by amplification with Tcz1 and Tcz2 were labelled with α^32^P-dATP using the Primer Labelling System (Invitrogen). The radiolabelled probes were purified in a Sephadex G50 glass wool column, yielding 10^7^ cpm/μg of probe, which was used at a concentration of 1-2 × 10^6^ cpm/mL of hybridisation solution. The membrane was blocked for 3 h at 65°C with salmon sperm DNA (100 μg/mL) in pre-hybridisation solution containing PEG 800 (10%), SSPE (1.5%), and SDS (7%). Then, the denatured, radiolabelled probes were incubated with the membrane for 12 h at 65°C for hybridisation. After washing in increasingly stringent solutions to remove unbound probe, the membrane was wrapped in plastic film for cassette exposure to X-ray film overnight at −80°C.

*Enzyme-linked immunosorbent assay (ELISA)* - ELISA was used to detect soluble *T. cruzi* antigens (1 μg/100 μL in 0.1 M carbonate buffer, pH 9.6) on coated micro-plate wells. Sera were diluted 1:100 and incubated with the antigens in the wells as previously described (Ribeiro et al. 2016). The test and control sera assays were run in triplicate, and the OD results reported are the means ± standard deviation. The cut-off, determined with a bank of serum samples, was an absorbance ≥ 0.150.

*Indirect immunofluorescence test (IFI)* - For the IFI, mouse serum dilutions (20 μL) in PBS (pH 7.4) were incubated with glass slides smeared with formalin-killed *T. cruzi* Berenice epimastigotes (Ribeiro et al. 2016).

*Immunohistochemistry* - Tissue sections from *T. cruzi-*infected and naïve uninfected control mice were fixed in formalin and processed for immunohistochemistry (Coventry et al. 1995). The paraffin-embedded tissues were cut into 4-μm thick sections and were treated with several changes of xylene and gradually hydrated in a graded series of ethanol (100% to 70%; 1 min each). The tissue sections were incubated with 0.05% saponin in distilled water at room temperature. After three washes, the tissue sections were blocked with 5% non-fat powdered milk for 45 min. Then, the slides were washed in 0.1 M PBS (pH 7.4) and incubated with a 1:20 dilution of serum from a chronically *T. cruzi-*infected mouse or a control mouse. After 2 h, the slides were washed in PBS three times for 5 min each and allowed to dry at room temperature before incubation with an anti-IgG monoclonal antibody labelled with peroxidase (Santa Cruz Biotechnology). The slides were washed in PBS three times for 3 min each and then counterstained with Harris hematoxylin for 30 s; washed with distilled water; dehydrated in a graded series of ethanol (70%, 80%, 90%, and 100%) for 1 min each; and mounted in buffered glycerine. The slides were examined under a bright field light microscope, and images were captured with an Olympus DP76 U-TVO-63XC micro camera with Cell Sens Dimension Software.

*Statistical analysis* - One-way ANOVA was used for group analyses, and Tukey's test was employed to compare the means and standard deviations of the experimental data with aid of GraphPad Prisma^®^ version 6 software. A p value less than 0.05 was considered statistically significant.

*Ethics* - The study, using a mouse model, was approved by the Faculty of Medicine Ethical Committee on Animal Research (protocol no. 10411/2011).

## RESULTS

Trypomastigotes were visualised in the peripheral blood of mice that received intraperitoneal inoculations of 1 × 10^5^ *T. cruzi* by direct microscopic examination until three weeks after infection (Supplementary data, Figure). After breeding, *T. cruzi* flagellates were observed in the blood cultures of four mice, two intraperitoneally infected males and two naïve female mating partners. Parasitological demonstration of protozoan trypomastigotes in the blood of 20% (2/10) of naïve uninfected mice after mating with infected partners suggested that these *T. cruzi* infections were acquired through sexual transmission.

**Fig. 1:**
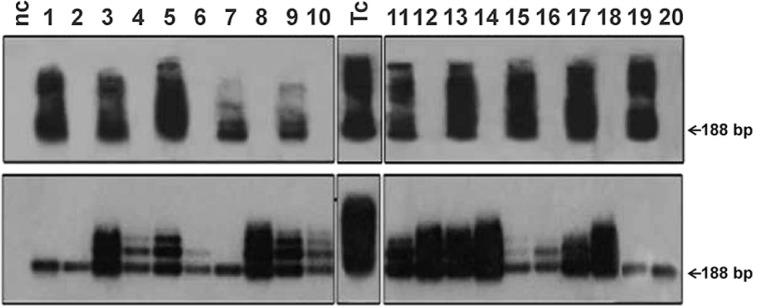
molecular demonstration of *Trypanosoma cruzi* infection in mice infected via sexual intercourse. The Tcz1/2 188-bp amplicon and catamers were detected with radiolabelled *T. cruzi-*specific nDNA probes. The top panel shows bands from intraperitoneally infected mice (odd numbers; males, 1-9; females, 11-19) before mating with uninfected animals. The bottom panel shows specific bands from mating partners after breeding (even numbers; females, 2-10; males, 12-20). Smears represent excess amplified product. NC: negative control; Tc: *T. cruzi-*positive control.

PCR was employed to detect a 188-bp amplicon of *T. cruzi* nDNA in blood samples. Fig. 1 shows the PCR results for *T. cruzi* nDNA detection in intraperitoneally infected males and females before breeding. These experiments were run in triplicate, and the specific nDNA bands were detected by hybridisation with a *T. cruzi-*specific radiolabelled probe. All male and female infected mice had the 188-bp nDNA band and catamers in their blood, whereas uninfected animals did not. Interestingly, after mating, all of the naïve uninfected male and female mice had the same 188 bp bands as their infected mates (Fig. 1). The demonstration of *T. cruzi* DNA in the blood of naïve uninfected females after breeding with infected males reinforces that notion that these infections were sexually transmitted.

**Fig. 2:**
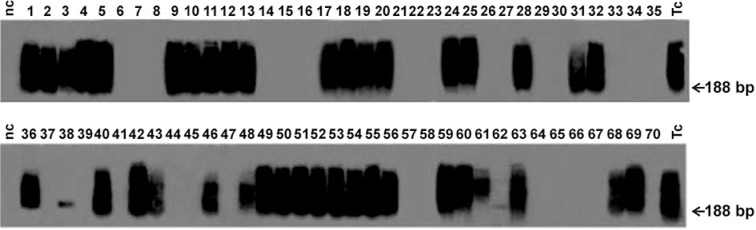
vertical transmission of *Trypanosoma cruzi* after sexual transmission in mice. Southern blotting of the 188-bp Tcz-amplified nDNA sequence by hybridisation with a specific radiolabelled probe. Top lanes are the offspring born after mating of infected males with naïve uninfected females. The bottom lanes are the offspring born after mating of infected females with naïve uninfected males. Notice that 16 mice in the top lane and 14 in the bottom lane (total 30/70, 51%) did not have the 188-bp nDNA band, therefore, they are uninfected. NC: negative control; Tc: *T. cruzi* DNA (positive control).

Moreover, the PCR results for progeny born to newly infected females mated with intraperitoneally infected males showed that 54% (19/35) of the offspring acquired the *T. cruzi* infection (Fig. 2). These data clearly demonstrate sexual transmission of the infection, because any congenital *T. cruzi* acquisition would require previous infection of the mother by mating. Fig. 2 (bottom lane) also shows vertical transmission of *T. cruzi* from intraperitoneally infected females (30 dpi) to 22 of their 35 progeny (63%) as evidenced by the presence of the 188-bp nDNA band. Furthermore, the PCR assays showed no statistical difference in the sexual transmission rates between males and females.

**Fig. 3:**
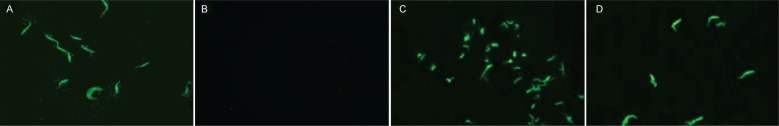
presence of *Trypanosoma cruzi-*specific IgG antibodies in the serum of sexually infected mice. Immunofluorescence of *T. cruzi* forms treated with mouse serum. The apple-green fluorescence was developed with a fluorescein-labelled secondary antibody against mouse anti-*T. cruzi* IgG. (A) Positive control, intraperitoneally infected mice. (B) Uninfected negative control. (C) Naïve male mice after mating with infected female mice. (D) Naïve female mice after mating with infected male mice.

ELISA and IFI assays were used to assess mouse serum for the presence of anti-*T. cruzi* IgG. The ELISA results showed specific IgG antibodies in the serum of all naïve females that were mated with *T. cruzi*-infected males, and in only three (60%) of the naïve male mice that were mated with *T. cruzi-*infected females. IFI as-says confirmed the results obtained by ELISA (Fig. 3). Of interest, the antibody titres from the male mice that were inoculated intraperitoneally (2,898 ± 1,415) were significantly different (p = 0.001) from those in the sera of mice that acquired the infections via sexual transmission (2,347 ± 1,048).

**Fig. 4:**
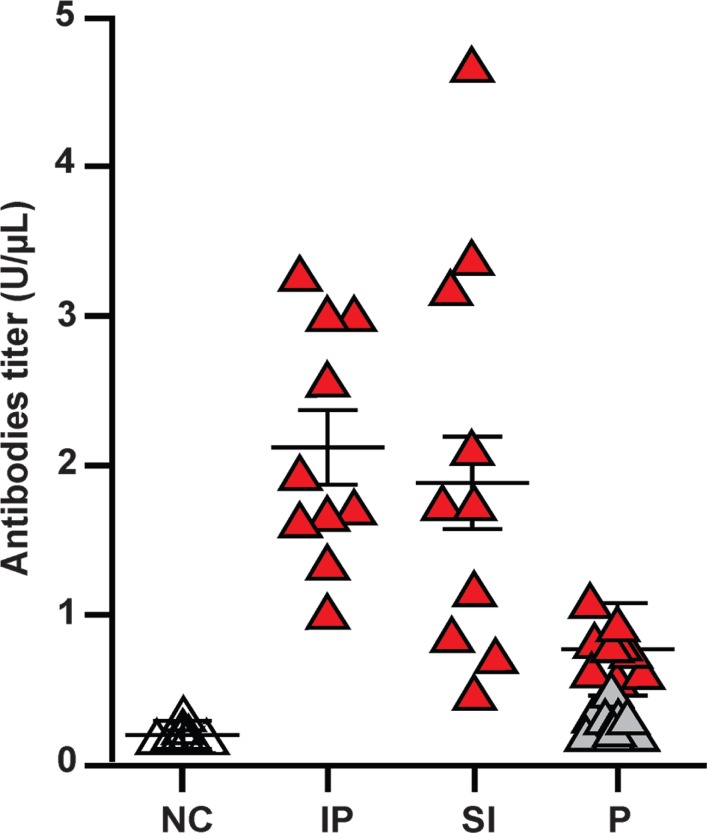
titration of anti-*Trypanosoma cruzi* antibodies. Antibody titres (IgG) in each group were determined by ELISA as the optical density at 490 nm (OD_490_). Test and control serum assays were run in triplicate. One-way ANOVA was used for group analyses, and Tukey's test was used to compare the means and standard deviations of experimental data. A p value less than 0.05 was considered significant. NC: negative control; IP: intraperitoneally infected mice; SI: sexually infected mice; P: progeny; black triangles: antibody titres below the cut off; red triangles: antibody titres above the cut off.

Animals infected by the intraperitoneal route showed antibody titres similar to those in animals infected through sexual intercourse, but were higher than the titres in progeny (Fig. 4). In fact, among the offspring showing nDNA bands, suggestive of vertically acquired infections, as few as 9 out of 41 (22%) had anti-*T. cruzi* antibodies. Plotting the ELISA values revealed significant differences between the negative controls and intraperitoneally infected mice (positive controls), and among the sexually infected mice (p < 0.05).

**Fig. 5:**
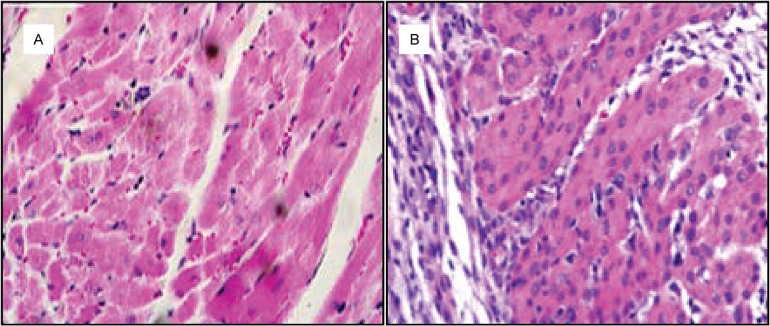
chagasic myocarditis in mice infected through the sexual route. (A) Negative control heart histology. (B) Histology of male mice infected through sexual intercourse. Notice the inflammatory lymphocyte infiltrates and lysis of target heart fibres. H&E stained sections, 400×.

An average of twelve tissue sections from each mouse in the mated couples and the control groups were subjected to a histopathological analysis for nests of *T. cruzi* amastigotes and to evaluate the inflammatory pathology in the heart, skeletal muscle, digestive system, and reproductive organs. No *T. cruzi* amastigote nests were observed in the tissues of mice in the chronic phase of the infection in the H&E-stained sections. However, very intense inflammatory infiltrates, with destruction of host tissues, were observed in mice infected by both the intraperitoneal and sexual routes (Fig. 5).

**Fig. 6:**
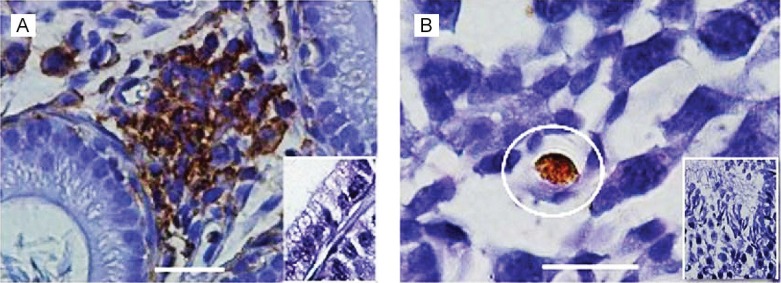
*Trypanosoma cruzi* forms in male sexual organs. Microphotographs showing (A) brownish immunoperoxidase-stained *T. cruzi* in the interstitial tissue of the epididymis, and (B) *T. cruzi* amastigotes nests in gonadal cells (circle). Inserts, negative control. Bars: 10 μM

Several authors have reported *T. cruzi* amastigote nests in the sexual organs of humans (Vianna 1911) and mice (Lenzi 1998, Carvalho et al. 2009). Here, tissue sections from the reproductive organs were obtained for microscopic examination, and the *T. cruzi* forms in the seminiferous tubules and epididymis are shown in Fig. 6. Immunohistochemistry enabled the identification of structures compatible with amastigote nests of *T. cruzi*, corroborating studies that suggested a tropism for the testes. These results could be explained by the capacity of the reproductive organs to tolerate *T. cruzi* infection without eliciting an inflammatory immune response, which is known as immune privilege (Goodnow et al. 1988).

## DISCUSSION

In this study, we inoculated male and female mice with *T. cruzi* trypomastigotes and sought to evaluate possible sexual transmission of the parasite from infected males to uninfected females and *vice-versa*. Direct microscopic parasitological examination revealed that all intraperitoneally infected mice had circulating trypomastigotes in the blood for three weeks after infection, which may explain why their naïve, uninfected mates readily acquired *T. cruzi* after sexual intercourse. However, only two males infected by the intraperitoneal route and two females infected by sexual intercourse had parasitemia, as detected by blood culture in the chronic phase of the infection.

Therefore, sexual transmission was evaluated by PCR using the Tcz1/2 primer set, which amplifies a 188-bp fragment of *T. cruzi* nDNA. The results of these PCR as-says showed that all male and female mice that mated with *T. cruzi-*inoculated mice acquired the infection. These nDNA-positive founders (F0) generated progeny. When the progeny mice reached six weeks of age, ~0.5 mL of blood was drawn via heart puncture. PCR showed that these founders had the 188-bp nDNA bands, indicating active *T. cruzi* infection. Therefore, the parasite was vertically transmitted to F1 progeny. Of the 70 F1 progeny, 41 (58.6%) were nDNA positive. Interestingly, of these mice with nDNA bands suggestive of vertically acquired infections, only 9 out of 41 (22%) had anti-*T. cruzi* antibodies.

The observed discrepancy between the results obtained for the parasite-specific antibody assay and the PCR analysis may be explained by the higher sensitivity of molecular tests over serological methods at the early stage of congenital infection. Furthermore, the differences in parasitaemia among infected mothers, which may be related to maternal immunity, may influence maternal-foetal transmission of the parasites and should be considered (Carlier et al. 2015). Another explanation is the possibility of immune tolerance to parasite antigens during embryonic development before maturation of the immune system, and sustained life-long by self-tolerance regulatory T cells (Sakaguchi et al. 1995, Ashour & Seif 2007).

In conclusion, our results demonstrate that sexual transmission of *T. cruzi* is a frequent event in mice, as most naïve animals became infected after mating with infected partners, which corroborates the findings of Ribeiro et al. (2016). Host-to-host transmission via coitus is possible because *T. cruzi* can survive in almost all body tissues, including reproductive tissues. A highly sensitive immune-peroxidase technique (Coventry et al. 1995) was used to detect clumps of *T. cruzi* in the reproductive tissues of infected males, and parasites were observed in gonadal blast cells, the lumen of the seminiferous tubules, and the interstitial epididymis. In agreement with these findings, colonisation of the testes, seminiferous tubules, and ovaries of *T. cruzi*-infected mice was also demonstrated (Lenzi et al. 1998, Carvalho et al. 2009). Furthermore, it has been shown that semen is adequate vehicle for the transport of *T. cruzi* (Carvalho et al. 2009), and this should also be true for vaginal secretions.

Small rodents are a group with huge potential for *T. cruzi* dissemination. Approximately 43% of all South America mammals are rodents (Teixeira et al. 2011), and they cohabit the same ecosystems as the parasite. Several studies have examined the rates of *T. cruzi* infection in wild rodents (Galuppo et al. 2009, Orozco et al. 2013). The high prevalence rates among rodents helps to sustain the enzootic transmission cycle of *T. cruzi* (Jansen et al. 2015).

These data suggest that *T. cruzi* sexual transmission may contribute to the spread of this flagellate among free-ranging mammals, representing a new enzootic scenario, in which rodents participate in a non-vectorial route of transmission. Thus, these animals can act as both reservoirs and vectors, and *T. cruzi* acquisition through sex intercourse could be the ancient route of transmission.

Laboratory mice provide an excellent model for understanding *T. cruzi* sexual transmission, and the results can be correlated with findings in wild rodents. Thus, they can be used in future studies to evaluate whether distinct *T. cruzi* DTUs can affect the parasite's ability to be transmitted by sexual contact. Further investigations are needed to determine if *T. cruzi* infections can affect an animal's reproductive health. Epidemiological surveys in areas with a low incidence of triatomines may help to understand the importance of this route of transmission in wild mammals.
