# Supplementary figures and images for: Can sexual transmission support the enzootic cycle of Trypanosoma cruzi?
Source: Mem Inst Oswaldo Cruz. 2018 Jan;113(1):3–8. doi: 10.1590/0074-02760170025 (PMC5719536; doi:10.1590/0074-02760170025)

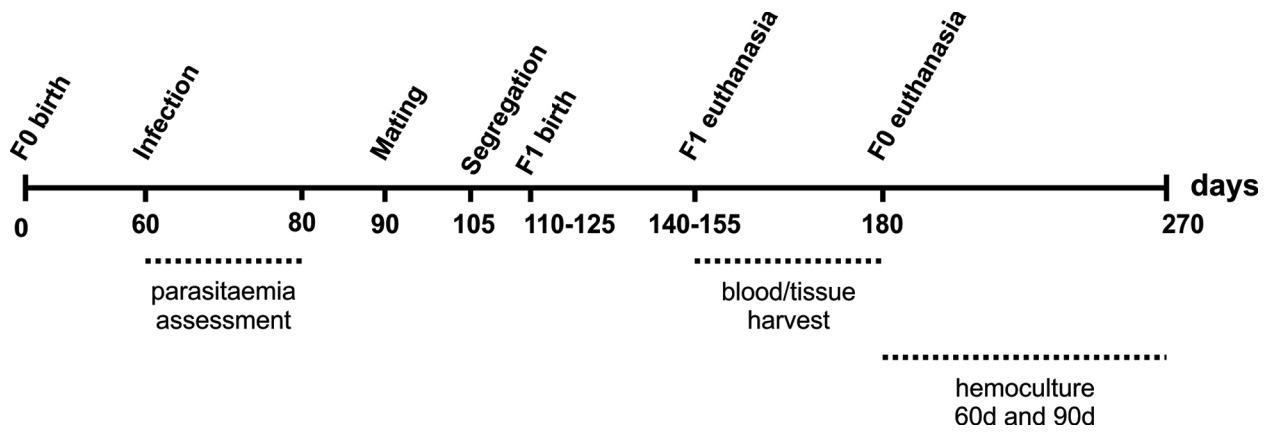

Timeline of the experiment.

Supplement: Supplementary file 1 [file 0074-0276-mioc-113-01-0003-Suppl01.pdf]
